# The Institutional Worker

**Published:** 1912-05-04

**Authors:** 


THE
### INSTITUTIONAL WORKER
**Being a Special Supplement to "The Hospital"**

### Editor's Notices.

**Contributions:**

Contributions should be written, or preferably typed, on one side of the paper only, and all articles sent in are accepted upon the distinct understanding that they are forwarded to THE HOSPITAL only.

The Editor cannot undertake to return MSS. not used, but when a stamped directed envelope is enclosed the MSS. may be returned if a special request is made.

Accepted articles and paragraphs of news will be paid for after publication at the scale rate.

**Address:**

To prevent delay all contributions and letters on editorial business must be addressed exclusively to the Editor, "The Hospital," 29 Southampton Street, Strand, London, W.C. It is important that this regulation shall be strictly observed.

**Correspondence:**

Correspondence on all subjects is invited. The name and address of correspondents must be given as a guarantee of good faith, but not necessarily for publication.

**Special Articles:**

Special articles are invited, and questions, inquiries, and paragraphs upon all matters relating to the work, administration, and management of general, special, mental, fever, cottage and convalescent hospitals, sanatoria, homes, institutions, societies and organisations for the treatment or care of the sick, injured and dependants of all classes. Every matter affecting the interests and welfare of the staff of all grades working in these institutions will receive special consideration.

**Administrative Medicine, Research and Items of News:**

Special terms will be paid for approved articles on subjects in this wide field by experts who have made some section of it their special study and interest. We wish to give prominence to every movement tending to advance accuracy of diagnosis, efficiency in treatment, and the development of modern methods to eradicate disease and promote the welfare of the suffering.

**A Bureau of Information:**

We invite inquiries and applications for information and help in providing for that numerous class of sufferers who require special care or house-room which they cannot obtain in their own homes or secure for themselves. We cannot, however, prescribe, or recommend practitioners.

**Books for Review:**

Publishers are particularly requested to send advance proofs of any new books of importance whenever possible, as the Editor has made arrangements to publish immediate reviews on a new plan.

**Photographs, Plans, Blocks, and Illustrations:**

It is requested that wherever possible MSS. may be accompanied by illustrations in any of the above forms. The name of the sender and of the article to which it belongs should be written on the back of each photograph, drawing, or block for purposes of identification.

**Local Papers:**

Newspapers containing reports or news paragraphs should be marked and addressed to the Sub-Editor.

**The Coupon System:**

A coupon will be found at the bottom of the third inside page of the cover each week. This coupon must be attached to every question or inquiry to which an answer is desired in THE HOSPITAL.

### Out of Print.

Last week the entire Edition of the Special Number of THE HOSPITAL was sold out by Saturday morning, and, although many inquiries have since been received for this important issue, it is now "out of print." This is undoubtedly the result of the high appreciation in which THE HOSPITAL is regarded by Executive Officials and Institutional Workers of all grades throughout the country. There is no Journal that deals so adequately and comprehensively with Institutional affairs, and consequently each issue of THE HOSPITAL is eagerly awaited by all keen and progressive Officers in the Charitable, Poor-Law, and Municipal Services. It is essential, therefore, that everyone who desires to receive THE HOSPITAL regularly should place their order permanently with their Newsagent and Bookseller or send a subscription direct to the Publishing Offices at the undermentioned rates :

**Subscriptions,** which may commence at any time, are payable in advance. Cheques and Post-Office Orders should be crossed London County and Westminster Bank, Covent Garden Branch, and made payable to the Manager of THE HOSPITAL.

RATES OF SUBSCRIPTION (payable in advance).

UNITED KINGDOM.

| | | |
|---|---|---|
| Three Months | (including Postage) | 2s. 0d. |
| Six Months | do. | 4s. 0d. |
| Twelve Months | do. | 6s. 6d. |

FOREIGN AND COLONIAL.

| | | |
|---|---|---|
| Three Months | (including Postage) | 2s. 6d. |
| Six Months | do. | 5s. 0d. |
| Twelve Months | do. | 8s. 8d. |

*SUBSCRIPTION FORM.*

*Please send me* THE HOSPITAL *for*.....................*months,*

*commencing with your issue of*................................, *for*

*which please find enclosed*............................

*Name*..........................................................

*Address* ..........................................................

..........................................................

*Date*..........................................

*To*.................................*Newsagent.*

### Manager's Notices.

**All letters on business matters must be addressed to The Manager, "The Hospital," The Hospital Building, 28 and 29 Southampton Street, London, W.C.**

**Advertisements:**

To insure insertion, all advertisements for THE HOSPITAL must reach the Manager not later than Wednesday morning in each week. For scale of charges see pages v. and 2.

**Special Rates** will be quoted for those Institutions that agree to send all their vacancy, contract, and public notices to THE HOSPITAL each year, so as to make it a file of such announcements for ready reference.

**Remittances:**

**It is especially requested that remittances be made by cheque or Postal Order, and in halfpenny stamps only when the amount is under sixpence.**

### OFFICIAL APPOINTMENTS VACANT.

### Poor-Law Vacancies.

Assistant Labour Master (£26), Camberwell Guardians. Mr. C. S. Stevens, C. to G., 29 Peckham Road, S.E. May 6.

Assistant Laundress (£20), Wirral Union. Mr. C. H. Spelman, Master of the Workhouse, Clatterbridge, nr. Birkenhead. May 4.

Assistant Male Lunatic Attendant (£30), Stoke-on-Trent Union. Mr. T. Wood, C. to G., Stoke on-Trent. May 7.

Assistant Nurse (£20), Ruthin Union. Mr. R. H. Roberts, C. to G., Ruthin. May 4.

Assistant Nurse (£27 10s.), Shardlow Union. Mr. J. W. Newbold, C. to G., Derby. May 6.

Assistant Nurse (£20), Hungerford and Ramsbury Union. Mr. H. D'O. W. Astley, C. to G., Hungerford. May 6.

Assistant Nurse (£25), Grimsby Union. Mr. J. F. Wintringham, C. to G., Grimsby. May 9.

Assistant Nurse (£25), Pontypridd Union. Mr. W. Spickett, C. to G., Pontypridd. May 9.

Assistant Nurse (£25), Solihull Union. F. L. Thompson, C. to G., Union Offices, Council House, Sparkhill, Birmingham. May 14.

Charge Nurse (£35), Bromley Union. Mr. E. Haslehurst, C. to G., Bromley, Kent. May 6.

Charge Nurse (£30), Keighley Union. Mr. S. Green, C. to G., Keighley. May 6.

Charge Nurse (£30), Holborn Union. Matron, Workhouse, Mitcham.

Charge Nurse (£35), Kingston-upon-Hull Guardians. R. H. Winter, C. to G., St. Mary's Chambers, Lowgate, Hull. May 14.

Charge Nurse (£30), Dudley Union. Mr. E. Allen, C. to G., Dudley. May 8.

Charge Nurses (2) (£36), Grimsby Union. Mr. J. F. Wintringham, C. to G., Grimsby. May 9.

Charge or Staff Nurse (£35), Macclesfield Union. F. May, C. to G., Union Offices, Macclesfield. May 13.

Cook (£22), Martley Union. Matron of the Workhouse, Martley, nr. Worcester. May 9.

Cook-General (£20), Goole Union. Mr. R. Haldenby, C. to G., Goole. May 7.

Enquiry Officer, etc. (25s. weekly), Whitechapel Union. Mr. F. J. Tootell, C. to G., 74 Vallance Road, N.E. May 9.

Female Attendant (£20), Salford Union. Mr. F. Townson, C. to G., Salford. May 8.

Female Attendant (12s. weekly), Bermondsey Guardians. Mr. E. Pitts Fenton, C. to G., 283 Tooley Street, S.E. May 6.

Female Teacher (£55), Southampton Guardians. Mr. A. J. Walden, C. to G., Southampton. May 4.

General Assistant to Matron (£20), Reigate Guardians, Mr. F. C. Morrison, C. to G., Reigate. May 14.

Head Laundress (£35), Edmonton Union. Mr. F. Shelton, C. to G., White Hart Lane, Tottenham. May 4.

Head Nurse (£30), Falmouth Union. Mr. R. N. Rogers, C. to G., Falmouth. May 7.

Nurses (£30), Winchester Union. F. Faithfull, C. to G., 105 High Street, Winchester. May 18.

Infants' Attendant (£25), Reigate Guardians. Mr. F. C. Morrison, C. to G., Reigate. May 14.

Labour Master (£35), Fulham Guardians. Mr. E. J. Mott, C. to G., 129 Fulham Palace Road, W. May 6.

Labour Master (£34), Hammersmith Guardians. The C. to G., 206 Goldhawk Road, Shepherd's Bush, W. May 6.

Laundress (£25), Fareham Union. Mr. A. Laker, C. to G., Fareham. May 10.

Male Nurse (£30), Godstone Union. G. Sneezum, Master of the Workhouse, Bletchingley.

Master and Matron (£60 and £45), Tamworth Union. Mr. J. H. Dewes, C. to G., Tamworth. May 4.

Mental Attendants (married couple) (29s. weekly, non-resident), Rochford Union. Mr. F. Gregson, C. to G., Southend-on-Sea. May 6.

Nurse (£30), Ruthin Union. Mr. R. H. Roberts, C. to G., Ruthin. May 4.

Nurse (£30), Foleshill Union. Mr. A. P. Oswin, C. to G., Foleshill. May 7.

Nurse (£30), Ormskirk Union. A Dickinson, C. to G., Union Offices, Ormskirk. May 8.

Nurse (£30), Barnet Union. A. Wilshire, C. to G., Union Offices, Barnet. May 13.

Nurse (£30), Isle of Thanet Union. C. Taylor, C. to G., Minster, nr. Ramsgate. May 23.

Nurse (£30), Knighton Union. E. H. Deacon, C. to G., Union Offices, Knighton. May 15.

Nurses (2) (£25 or £30 according to qualifications), Winchester Union. Mr. F. Faithfull, C. to G., Winchester. May 13.

Seamstress (£26), Edmonton Union. Mr. F. Shelton, C. to G., White Hart Lane, Tottenham. May 4.

Second Laundrymaid (£18), Edmonton Union. Mr. F. Shelton, C. to G., White Hart Lane, Tottenham. May 4.

Staff Nurses (£25), Salford Union. Mr. F. Townson, C. to G., Salford. May 8.

Superintendent and Matron of Children's Home (£90 and £60), Ipswich Guardians. Mr. L. W. Greenhalgh, C. to G., Ipswich. May 8.

Superintendent Nurse (£60), Chesterfield Union. Mr. R. F. Hartwright, C. to G., Chesterfield. May 10.

### Institutional and Administrative Vacancies.

Assistant Matron (£40), Preston Royal Infirmary. W. Davies, Secretary, 5 Winckley Street, Preston. May 13.

Matron (£100), City of London Hospital for Diseases of the Chest, Victoria Park, E. G. Watts, Secretary. May 13.

Matron (£50), Yeovil District Hospital. F. W. Mayo, Hon. Sec., Swallowcliffe, Yeovil. May 10.

Matron (£90), Herefordshire General Hospital. W. A. W. Price, Secretary. May 6.

Sister (£35), Oldham Royal Infirmary. E. Lionel Blake, Superintendent-Secretary. May 8.

